# Changes in Inflammatory Cytokines After Chronic Hepatitis C Treatment Among People Living With HIV

**DOI:** 10.1093/ofid/ofad623

**Published:** 2024-01-04

**Authors:** Hamidreza Karimi-Sari, Damani A Piggott, Eileen P Scully, Kathleen Ward, Catherine G Sutcliffe, Mark Sulkowski, Oluwaseun Falade-Nwulia

**Affiliations:** Division of Infectious Diseases, Johns Hopkins University School of Medicine, Baltimore, Maryland, USA; Division of Infectious Diseases, Johns Hopkins University School of Medicine, Baltimore, Maryland, USA; Department of Epidemiology, Johns Hopkins Bloomberg School of Public Health, Baltimore, Maryland, USA; Division of Infectious Diseases, Johns Hopkins University School of Medicine, Baltimore, Maryland, USA; Division of Infectious Diseases, Johns Hopkins University School of Medicine, Baltimore, Maryland, USA; Department of Epidemiology, Johns Hopkins Bloomberg School of Public Health, Baltimore, Maryland, USA; Department of International Health, Johns Hopkins Bloomberg School of Public Health, Baltimore, Maryland, USA; Division of Infectious Diseases, Johns Hopkins University School of Medicine, Baltimore, Maryland, USA; Division of Infectious Diseases, Johns Hopkins University School of Medicine, Baltimore, Maryland, USA

**Keywords:** chronic hepatitis C, direct-acting antivirals, HIV, mediators of inflammation

## Abstract

We aimed to evaluate the effect of hepatitis C virus cure on serum inflammatory markers among people with HIV. Among 127 people with HIV, serum alanine aminotransferase, soluble tumor necrosis factor receptor 1, and inflammatory index score were significantly lower at the 24-week time point in patients who achieved sustained virologic response as compared with those who did not.

Among people with HIV (PWH), markers of immune dysregulation and heightened inflammation, such as interleukin 6 (IL-6) and soluble tumor necrosis factor receptor I (sTNFR-I), are associated with adverse clinical outcomes, including cardiovascular disease, cognitive decline, frailty, and mortality [1–3]. Independently, chronic hepatitis C virus (HCV) infection is also linked to elevated levels of systemic inflammatory markers, including IL-6 [4], which may contribute to the pathogenesis of liver fibrosis and hepatocellular carcinoma [5, 6]. Coinfection with both viruses has been associated with higher levels of IL-6 and sTNFR-I when compared with HIV monoinfection. Among PWH, HCV cure following direct-acting antiviral (DAA) treatment has been associated with a reduction in markers of T-cell activation, but the reported effect on markers of systemic inflammation has been variable [3, 7]. Among adults without HIV, inflammatory index score (IIS; derived from levels of IL-6 and sTNFR-I) was a better predictor of 10-year mortality than IL-6 or sTNFR-I [8]. Defining the impact of HCV cure on residual inflammation and the associated complications and comorbidities in PWH remains an important clinical and scientific question. In this study, we evaluated the impact of HCV cure following DAA treatment on alanine aminotransferase (ALT), IL-6, sTNFR-I, and IIS in people with HCV/HIV coinfection.

## METHODS

The CHAMPS study (NCT02402218) was a randomized controlled trial that enrolled patients with HIV and untreated HCV who were receiving care at the Johns Hopkins HIV clinic. Participants were randomized to usual care, usual care plus cash incentives, and usual care plus peer mentor arms to evaluate HCV treatment initiation. Enrolled participants were ≥18 years old with a CD4 count >100 cells/mm^3^, an estimated glomerular filtration rate ≥30 mL/min/1.73 m^2^, and no evidence of hepatocellular carcinoma or decompensated liver disease. The majority of participants initiated treatment with ledipasvir/sofosbuvir, received 12 weeks of therapy, and were assessed at post treatment week 12; participants who did not start therapy were followed up 24 weeks after treatment noninitiation [9].

Quantitative HCV RNA (COBAS TaqMan HCV Test version 2.0; Roche Molecular Systems Inc) was performed before and ≥24 weeks after HCV treatment initiation or postenrollment. CD4 cell count and HIV RNA levels were measured at screening, and liver elastography was performed (FibroScan 502 Touch; Echosens North America). At enrollment, drug and alcohol use was assessed with the Alcohol Use Disorders Identification Test and by urine toxicology (US Drug Testing Laboratories). Hazardous alcohol use was defined as a test score ≥8 for men and ≥4 for women [10].

Participants were categorized by HCV RNA status as having achieved or not achieved sustained virologic response (SVR; HCV RNA <15 IU/L) at ≥12 weeks after HCV treatment completion. Serum IL-6 and sTNFR-I were measured with commercially available enzyme-linked immunosorbent assay kits on samples stored at −80 °C collected before and ≥24 weeks after HCV treatment initiation or noninitiation. The IIS was calculated from the IL-6 and sTNFR-I measurements as previously described and validated [11]. HIV RNA ≤20 was considered undetectable.

Data were analyzed with SPSS (version 22; IBM Corp). Participant characteristics were quantitatively described. Patient groups with and without SVR were compared with an independent *t* test or Mann-Whitney *U* test for continuous variables and a chi-square or Fisher exact test for categorical variables. Patient-level paired comparisons of baseline and 24-week markers were compared with the Wilcoxon signed rank test. Linear correlations between variables were assessed with the Spearman rho test, and the correlation coefficient (*r*) and significance level (*P* value) were reported. *P* < .05 was considered statistically significant. Figures were generated by Prism software (version 8; GraphPad).

### Patient Consent Statement

The study was approved by the Johns Hopkins Medicine Institutional Review Board and conducted following provisions of the Declaration of Helsinki and good clinical practice guidelines. All participants provided written informed consent.

## RESULTS

Among 127 participants (99 SVR and 28 non-SVR) with baseline and follow-up blood specimens available, 75 (59%) were male, 116 (91%) were African American, and the median age was 54.0 years (IQR, 50.0–59.0; Table 1). At enrollment, most participants (97%) were receiving antiretroviral therapy and had an undetectable HIV viral load (68%). Most participants were infected with HCV genotype 1a (76%), and 11.5% had a liver fibrosis score >12 kPa on transient elastography, consistent with cirrhosis. There were no significant differences in patient demographics, substance use, hazardous alcohol use, HIV antiretroviral use, HIV viral suppression, HCV genotype, and urine toxicology results at baseline between patients who did and did not achieve SVR (Table 1).

**Table 1.** Characteristics of Study Participants at Enrollment and 24 Weeks After Treatment Initiation/Noninitiation in Total and Among People With and Without SVR

Characteristic	Total (n = 127)	SVR (n = 99)	No SVR (n = 28)	*P* Value
Age, y	54.0 (50.0–59.0)	54.0 (50.0–59.0)	53.5 (49.25–59.0)	.663^a^
Male	75 (59.1)	56 (56.6)	19 (67.9)	.283^b^
African American	116 (91.3)	91 (91.9)	25 (89.3)	.662^b^
Positive urine toxicology for cocaine or heroin	56 (45.9)	43 (44.8)	13 (50.0)	.638^b^
Risky alcohol use, AUDIT ≥8 (men), ≥4 (women)	34 (26.8)	26 (26.3)	8 (28.6)	.808^b^
On antiretroviral therapy	124 (97.6)	97 (98.0)	27 (96.4)	.530^c^
Undetectable HIV RNA	83 (68.0)	68 (71.6)	15 (55.6)	.115^b^
CD4 count, cells/mm^3^	530.0 (354.0–796.0)	503.0 (354.0–809.0)	545.5 (341.2–718.7)	.868^d^
HCV genotype 1a	97 (76.4)	75 (75.8)	22 (78.6)	.757^b^
Liver fibrosis score, kPa				.604^b^
≤8	82 (67.2)	66 (69.5)	16 (59.3)	
>8 to <12	26 (21.3)	19 (20.0)	7 (25.9)	
≥12	14 (11.5)	10 (10.5)	4 (14.8)	
Baseline HCV RNA, 10^4^ IU/mL	393.0 (90.0–957.0)	313.0 (85.8–908.0)	503.5 (144.7–1552.5)	.248^d^
ALT, IU/L				
Baseline	33.0 (21.0–50.0)	33.0 (21.0–46.2)	33.5 (20.2–65.0)	.540^d^
After 24 wk	14.0 (11.0–20.5)	12.0 (10.0–16.25)	29.0 (27.0–45.0)	**<.001** ^d^
ΔALT	−14.5 (−30.5 to −5.0)	−19.0 (−36.5 to −9.5)	−1.0 (−13.0 to 6.0)	**<.001** ^d^
IL-6, pg/mL				
Baseline	2.28 (1.43–3.91)	2.05 (1.40–3.16)	3.21 (1.49–5.97)	**.042** ^d^
After 24 wk	2.27 (1.30–3.52)	2.16 (1.13–3.71)	2.67 (1.64–3.39)	.120^d^
ΔIL-6	−0.26 (−1.07 to 0.36)	−0.21 (−1.00 to 0.36)	−0.31 (−2.69 to 0.56)	.444^d^
sTNFR-I, pg/mL				
Baseline	1649.3 (1274.6–2159.0)	1577.8 (1222.2–2113.5)	1741.5 (1277.5–2786.5)	.402^d^
After 24 wk	1438.5 (1112.5–1828.4)	1397.4 (1060.1–1768.6)	1657.4 (1375.4–2003.8)	**.033** ^d^
ΔsTNFR-I	−228.7 (−434.6 to 1.87)	−277.0 (−444.7 to −41.4)	5.99 (−417.5 to 176.0)	**.021** ^d^
Inflammatory index score				
Baseline	2.268 (2.159–2.425)	2.259 (2.151–2.381)	2.372 (2.190–2.496)	.114^a^
After 24 wk	2.230 (2.071–2.358)	2.200 (2.039–2.334)	2.298 (2.181–2.438)	**.040** ^a^
ΔInflammatory index score	−0.070 (−0.153 to 0.036)	−0.071 (−0.153 to 0.029)	−0.052 (−0.153 to 0.108)	.331^d^

Data are presented as No. (%) or median (IQR). *P* < .05 indicated in bold.

Abbreviations: ALT, alanine aminotransferase; AUDIT, Alcohol Use Disorders Identification Test; HCV, hepatitis C virus; IL-6, interleukin 6; sTNFR-I; soluble tumor necrosis factor receptor I; SVR, sustained virologic response.

^a^Independent *t* test.

^b^Chi-square test.

^c^Fisher exact test.

^d^Mann-Whitney *U* test.

At baseline, patients with and without SVR had similar levels of serum ALT, sTNFR-I, and IIS (*P* > .05). After 24 weeks, median serum ALT (12.0 vs 29.0 IU/L, *P* < .001), sTNFR-I (1397.4 vs 1657.4 pg/mL, *P* = .033), and IIS (2.19 vs 2.29, *P* = .040) were significantly lower in patients who achieved SVR vs the patients who did not achieve SVR. Median serum IL-6 was lower among people who achieved SVR at baseline (2.05 vs 3.21 pg/mL, *P* = .042). IL-6 levels after 24 weeks (2.16 vs 2.67 pg/mL, *P* = .120) and change in serum IL-6 (−0.21 vs −0.31 pg/mL, *P* = .444) were comparable between groups (Table 1). In paired analysis of baseline and 24-week markers, significant reductions in ALT, sTNFR-I, and IIS levels (*P* < .001) and a trend toward a decrease in serum IL-6 (*P* = .077) were observed among PWH who achieved SVR. There were no significant changes in these markers in people who did not achieve SVR (*P* > .05).

**Figure 1.**
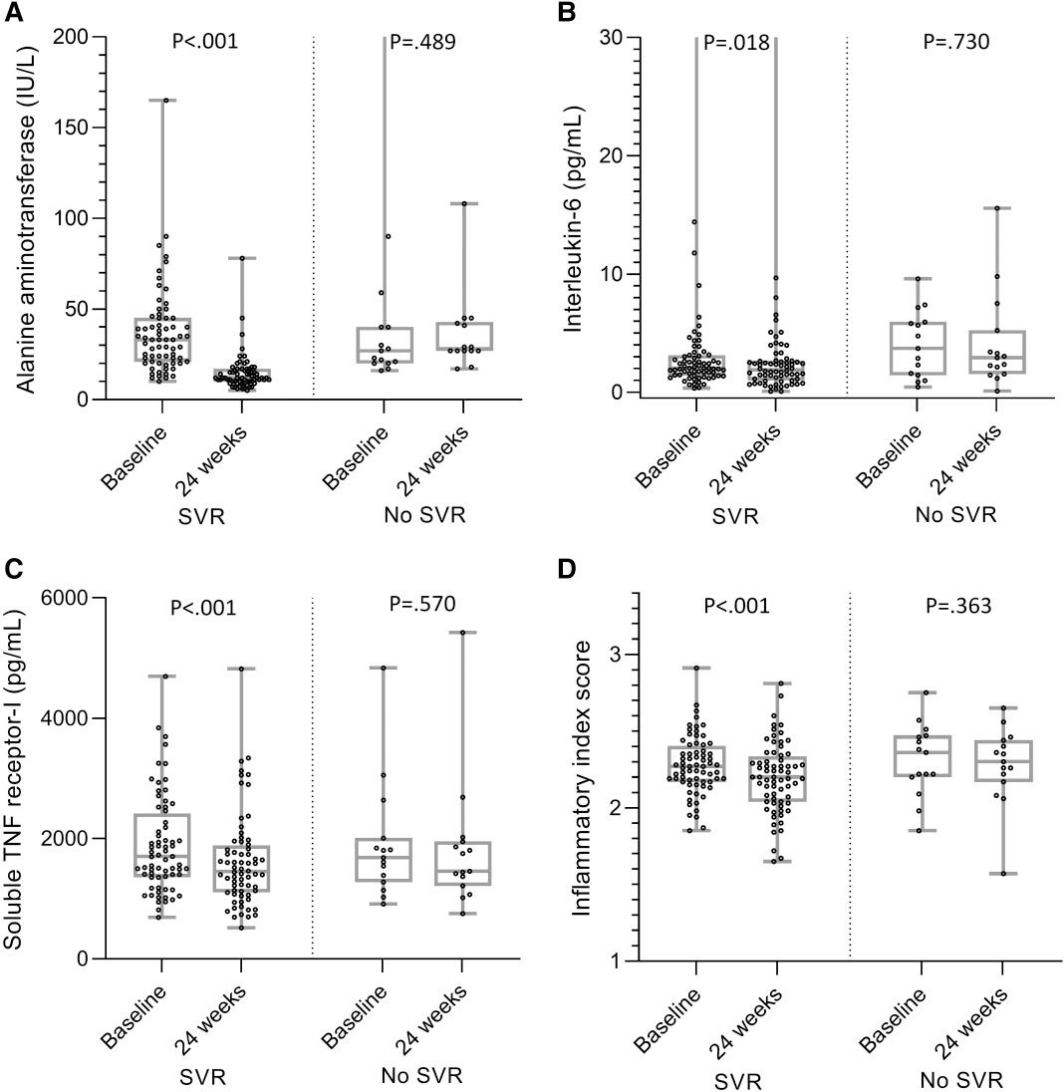
Levels at baseline and 24 weeks after treatment initiation/noninitiation: *A*, alanine aminotransferase; *B*, interleukin 6; *C*, soluble TNF receptor I. *D*, Inflammatory index score in a subset of people with HIV without detectable HIV viremia (n = 83): with SVR (n = 68) and without SVR (n = 15). Data are presented as median (line), IQR (box), and minimum and maximum values (bars). Wilcoxon signed rank test was used for the paired comparison. SVR, sustained virologic response; TNF, tumor necrosis factor.

In sensitivity analysis conducted among individuals with undetectable HIV viremia (n = 83), patients with (n = 68) and without (n = 15) SVR had similar baseline levels of serum ALT, IL-6, sTNFR-I, and IIS (*P* > .05). After 24 weeks, median (IQR) serum ALT was significantly lower in patients who achieved SVR as compared with those who did not (12.0 [10.0–17.0] vs 28.0 [27.0–42.7] IU/L, *P* < .001). Median IL-6 was lower (1.90 [1.02–2.66] vs 2.92 [1.56–5.23], *P* = .082) and median reduction in sTNFR-1 was greater (−300.1 [−524.4 to −42.8] vs 30.8 [−366.3 to 168.2], *P* = .058) in the group of PWH who achieved SVR vs the group that did not. Paired analyses of baseline and 24-week markers showed significant decreases in ALT (33.0 [21.0–45.0] to 12.0 [10.0–17.0], *P* < .001), IL-6 (2.04 [1.44–3.12] to 1.90 [1.02–2.66], *P* = .018), sTNFR-I (1699.7 [1358.4–2411.8] to 1450.0 [1106.8–1881.9], *P* < .001), and IIS (2.27 [2.17–2.40] to 2.20 [2.04–2.33], *P* < .001) among PWH who achieved SVR. There were no significant changes in people who did not achieve SVR (*P* > .05; Figure 1).

Across all 127 PWH included in the study sample, serum IL-6 was correlated with sTNFR-I at baseline (*r* = .375, *P* < .001) and after 24 weeks (*r* = .393, *P* < .001). There were no significant correlations between other markers (IL-6 and ALT, sTNFR-I and ALT) or between inflammatory markers (IL-6, sTNFR-I, or IIS) and HCV RNA at baseline and after 24 weeks (*P* > .05).

## DISCUSSION

In our analysis of inflammatory markers in PWH, HCV cure was associated with significant reductions in serum ALT and sTNFR-I and the composite IIS; among PWH with undetectable HIV, HCV cure was also associated with lower levels of IL-6. Overall, these data support the hypothesis that the cure of chronic HCV infection may be associated with reductions in inflammatory markers, consistent with other studies linking active coinfection to immune activation in PWH. While we did not assess the risk of long-term clinical outcomes in this study, curative HCV treatment reduces liver mortality and may prevent additional comorbidity by reducing systemic inflammation [7, 12, 13].

Other studies have demonstrated the impact of HCV cure on reduction in other markers predictive of non–AIDS-related complications after DAA therapy in people with HCV/HIV coinfection [14].

Piggott et al showed that lower IIS predicted less frailty progression, and reduction in IIS over time was associated with frailty recovery [11]. Higher levels of sTNFR-I/II over time were also associated with non–AIDS-related morbidities or death among PWH [15]. Garcia-Broncano et al reported higher levels of sTNFR-I with HCV/HIV coinfection as compared with PWH alone [12]. Other data among people with HCV monoinfection suggest that although HCV cure reduces inflammation [4], people with high levels of pretreatment and fewer changes in immune markers after treatment have an increased risk for hepatocellular carcinoma development [5, 16].

Although there was a significant correlation between IL-6 and sTNFR-I levels at baseline and after 24 weeks, we did not find a significant reduction in IL-6 levels in PWH who achieved SVR vs those who did not. This finding is consistent with that of Anthony et al and López-Cortés et al [3, 7] and may be due to a short follow-up time and heterogeneity of baseline IL-6 levels due to variable age, sex, weight, HIV viremia, and cirrhosis status [3, 12, 15]. Our finding of significant reduction of IL-6 among PWH with undetectable HIV RNA suggests that untreated HIV-associated inflammation may have masked the impact of HCV treatment and cure on residual inflammation.

Other data suggest that aging, HIV replication and/or HCV infection, and liver cirrhosis each contribute to heightened T-cell activation, leading to naive CD4+ T-cell lymphopenia, another marker of significant morbidity in PWH [17]. Partial reduction in certain indicators of T-cell activation after HCV cure has been postulated to be due to factors beyond HCV viremia, including HIV viremia [17, 18].

Our study has limitations: a small sample size, a relatively short follow-up period, and an inability to assess other markers that predict risk of non–AIDS-related complications (eg, T-cell activation) or extrahepatic manifestations of HCV infection (eg, cryoglobulinemia) due to a lack of these data.

Overall, HCV treatment with DAAs led to a reduction in liver (ALT) and systemic (sTNFR-I, IIS, IL-6) inflammation with a significant reduction in IL-6 observed among PWH with undetectable HIV viremia when compared with PWH with ongoing HCV infection. Further research is needed to determine the clinical relevance of these findings.

## Notes

***Author contributions*.** Study design (O. F.-N., M. S.), data acquisition (K. W., C. G. S., M. S.), data analysis (H. K.-S., O. F.-N.), manuscript writing (H. K.-S., O. F.-N.), critical revision of manuscript (D. A. P., E. P. S., K. W., C. G. S., M. S.), approving the final version of the manuscript (H. K.-S., D. A. P., E. P. S., K. W., C. G. S., M. S., O. F.-N.).

***Data availability*.** The corresponding author can provide the data upon request.

***Financial support*.** This work was supported by the National Institutes of Health (National Institute on Drug Abuse; grants R01DA16065, R37DA013806, U01DA036935, K24DA034621, K23DA041294); and the Johns Hopkins Institute for Clinical and Translational Research and the Johns Hopkins Center for AIDS Research.
